# Autistic Adults Show Intact Learning on a Visuospatial Serial Reaction Time Task

**DOI:** 10.1007/s10803-023-05894-y

**Published:** 2023-01-14

**Authors:** Isaac N. Treves, Jonathan Cannon, Eren Shin, Cindy E. Li, Lindsay Bungert, Amanda O’Brien, Annie Cardinaux, Pawan Sinha, John D. E. Gabrieli

**Affiliations:** 1 Department of Brain and Cognitive Sciences, Massachusetts Institute of Technology, 43 Vassar Street 46-4077, Cambridge, MA 02139 USA; 2 Hock E. Tan and K. Lisa Yang Center for Autism Research, Massachusetts Institute of Technology, Cambridge, USA; 3 Department of Psychology, Neuroscience, and Behaviour, McMaster University, Hamilton, ON Canada; 4 Program in Speech and Hearing, Bioscience and Technology, Harvard University, Cambridge, MA 02138 USA

**Keywords:** Procedural learning, SRT task, Prediction, Autism

## Abstract

Some theories have proposed that autistic individuals have difficulty learning predictive relationships. We tested this hypothesis using a serial reaction time task in which participants learned to predict the locations of a repeating sequence of target locations. We conducted a large-sample online study with 61 autistic and 71 neurotypical adults. The autistic group had slower overall reaction times, but demonstrated sequence-specific learning equivalent to the neurotypical group, consistent with other findings of typical procedural memory in autism. The neurotypical group, however, made significantly more prediction-related errors early in the experiment when the stimuli changed from repeated sequences to random locations, suggesting certain limited behavioural differences in the learning or utilization of predictive relationships for autistic adults.

**Supplementary Information:**

The online version contains supplementary material available at 10.1007/s10803-023-05894-y.

Autism spectrum disorder (ASD) is diagnosed on the basis of challenges with social communication and interaction and restricted, repetitive behaviors (American Psychiatric Association, 2013). Researchers have searched for atypical domain-general learning mechanisms behind these phenotypes in autism. Several such mechanisms have been proposed that center on the construct of prediction. According to the predictive impairment in autism (PIA) hypothesis (Sinha et al., 2014), the autism phenotype may stem from difficulties in detecting and learning statistical regularities over time that can be used to make predictions about the future (“contingencies”), especially when those relationships are weak or occur over longer time intervals. Such an impairment is hypothesized to give rise to social challenges and repetitive behaviors as it plays out over the course of development (Northrup, 2017). A related perspective suggests that the autism phenotype arises from differences in the “precision” or weight afforded to immediate expectations or predictions (Lawson et al., 2014). For example, predictions and the resulting prediction errors may be heavily and inflexibly weighted (Van de Cruys et al., 2014) resulting in the impression that certain settings are inherently unpredictable or “volatile” (Lawson et al., 2017). This impression and associated neuromodulation may make it difficult to detect and learn whatever weak but useful statistical tendencies exist in these settings, and may therefore give rise to learning difficulties similar to those proposed by the PIA hypothesis. These hypotheses have motivated a range of experiments related to prediction in autism that have, on the whole, supported the existence of certain domain-general prediction-related differences in autism (Cannon & O'Brien et al., 2021).

In contrast to these accounts of predictive learning differences in autism, however, researchers have found that procedural learning is generally intact in autism (Foti et al., 2015). Procedural learning is characterized by the learning of unconscious motor, perceptual, or cognitive patterns, which poses a challenge to prediction-related accounts of autism. A task that is especially well-suited to testing accounts of impaired predictive learning is the serial reaction time (SRT) task. In this task, participants respond to a stimulus that appears at one of four locations on a screen by pressing a corresponding button as quickly as possible. Unknown to the participants, the stimulus locations follow a repeating sequence with probabilistic or deterministic predictive relationships. As general reductions in reaction times may reflect task learning and not sequence-specific learning or prediction, blocks of random sequences of stimuli locations are inserted in the task. Sequence-specific learning is evidenced if participants respond faster to repeating sequences of stimuli than to random sequences of stimuli.

The SRT task is considered a procedural learning task because learning can occur without explicit memory for—or awareness of—the repeating sequence (Nissen & Bullemer, 1987). Individuals with declarative or explicit memory disorders have exhibited typical learning of the repeating sequence despite their amnesic disorders (Knopman & Nissen, 1987; Nissen & Bullemer, 1987; Reber & Squire, 1994). Neurotypical (NT) individuals have exhibited sequence learning despite being unaware of the sequence by self-report or by performance on explicit tests of memory for the sequence after the SRT task performance (Nissen & Bullemer, 1987; Reed & Johnson, 1994; Willingham et al., 1989).

Multiple studies of SRT have found that sequence-specific learning with repeated exposure to a sequence was comparable between ASD individuals and NT individuals (Barnes et al., 2008; Brown et al., 2010; Izadi-Najafabadi et al., 2015; Nemeth et al., 2010; Rybicki et al., 2021; Travers et al., 2010; Zwart et al., 2017, 2018; see Supp.Table 1). These findings are mostly from studies of children and adolescents, but results are consistent in adults (Rybicki et al., 2021; Zwart et al., 2017, 2018). Because sequence-specific learning can be conceptualized as procedural or implicit predictive learning of the location of the next stimulus, these results present a challenge to theories of a broadly atypical predictive learning in autism. One limitation of these studies is that typical learning in ASD is assumed based on the absence of a difference in sequence-specific learning between ASD and NT groups. Because these studies had modest sample sizes (15–30 participants per group), it is possible that the studies lacked the power to detect a difference.

Here we conducted an online study with 61 autistic and 71 neurotypical individuals using an SRT task; this was the largest SRT study with autistic participants to date and about twice the sample size of most prior studies. As preregistered, we assessed reaction time and error outcomes as dependent measures of learning and performance. We hypothesized that there would be no group differences in overall reaction time learning, but that subtler differences in trajectories of learning would perhaps be found, indicating possible avenues for reconciling evidence of intact SRT performance in ASD with evidence of differences in prediction.

## Methods

### Participants

Adults were recruited and consented to participate in this online research study, which consisted of an initial screening and multiple separate sessions of online behavioral testing. Autistic adults were recruited through the simons foundation powering autism research for knowledge (SPARK) database. The SPARK database currently consists of over 15,000 autistic adults with a reported clinical autism diagnosis and includes phenotypic as well as genetic data (The SPARK Consortium, 2018). A comparison group of NT participants was recruited through Prolific, an online portal to screen and recruit participants for online research (Peer et al., 2017). Forty-two percent of NT participants reported the US as their country of birth, thirty-nine percent reported the UK and eighteen percent listed other countries. Both ASD and NT groups had nearly equal numbers of males and females based on self-reported biological sex (Table 1).

**Table 1:** Demographics

	ASD			NT		
Measures	M	SD	Range	M	SD	Range
Age	28.14	5.62	20.08–45.58	30.10	7.30	18.00–45.10
NVIQ	28.37	3.49	20–35	29.25	2.93	20–34
AQ	31.9	7.17	16–46	19.56	7.53	4–34*
Sex	30F, 31M	NA	NA	35F, 36M	NA	NA

*NVIQ* non-verbal IQ, *AQ* autism quotient

*One outlier participant had an AQ score of 43

Exclusion criteria for both groups included any history of head trauma resulting in concussion, a history of seizures, uncorrected hearing impairments, colorblindness, and premature birth. We also excluded participants who were non-native English speakers. For the neurotypical group, we excluded any participants who reported any history of diagnosis of psychiatric, mood, or neurodevelopmental disorder, or use of medications for these conditions. Individuals with coexisting ADHD diagnoses were excluded from the neurotypical group, but not the ASD Group, and so analyses were run with and without ASD individuals with co-occurring ADHD.

### Sample Characterization

All participants completed the Test My Brain matrix reasoning subtest (Germine et al., 2012), a measure of nonverbal IQ that was previously validated and normed in a large online sample. Only those individuals who achieved a score of 20 or above (~ 2.75 standard deviations below the mean of 28.8) on the matrix reasoning subtest were included in the study; 13 autistic and three non-autistic individuals were screened out due to IQ scores lower than this threshold. To further characterize participants, we collected responses on the Autism Quotient, a 50-item self-report of autism symptomatology (Baron-Cohen et al., 2001). Screening and characterization questionnaire responses were collected via Qualtrics.

### Materials and Design

Our experimental session was preregistered at https://osf.io/v4j25. Participants were instructed to complete the session online from a computer in a quiet, distraction-free environment, and to use headphones that covered or rested inside both ears. Participants first consented, and then were directed to the serial reaction time task through the online experiment interface Pavlovia (www.pavlovia.org). Instructions and experiments were presented in full screen mode to minimize distractions. Task instructions were delivered to participants as on-screen text and concurrent audio of the same text read aloud. After the SRT experiment was finished, participants were redirected to Qualtrics for surveys and compensation.

### Serial Reaction Time Task

**Fig. 1:**
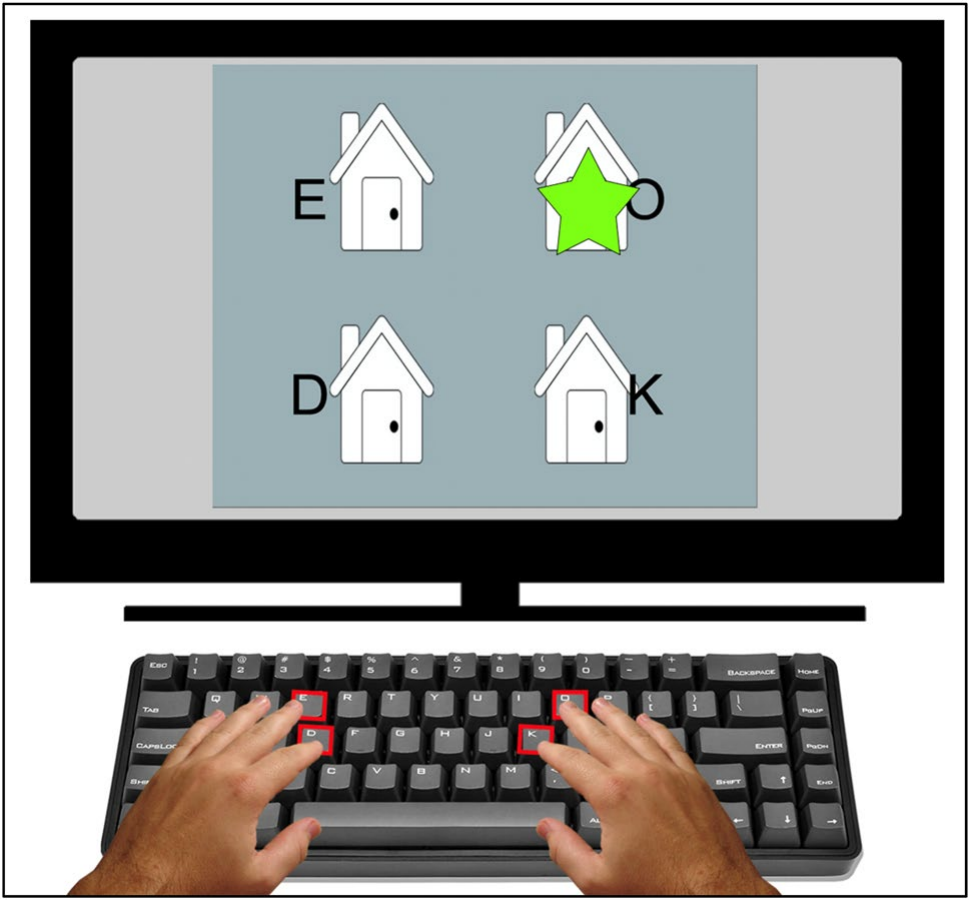
Example training block display, in which participants responded to stimuli arranged in a square layout. During each trial the green star appeared on a house. Letters were present only during the training block to facilitate mapping to responses. The response keys (E, O, D, K) and corresponding hand positions are also shown

Participants were asked to respond to a green star as it repeatedly appeared at one of four house locations on a screen (Fig. 1), identifying the location as quickly and accurately as possible. Images, text, and audio provided initial instructions related to finger position (Fig. 1). Participants were instructed to place their index fingers on the lower keys (D and K) and middle fingers on the upper keys (E and O). The keys spatially corresponded to the locations of the houses (e.g., E is the top-left key and corresponds to the top-left house). Each key press response triggered the presentation of the subsequent star, with a response-stimulus interval (RSI) of 120 ms. This rectangular array differs from the linear array used typically in SRT designs, but procedural learning has still been observed (Willingham et al., 2000; Jimenez et al., 2006).

In the experiment, there were “repeating” and “random” blocks. In repeating blocks, the star followed a deterministic 12-item sequence, and in random blocks the star followed a random sequence. The E, O, D, K keys correspond to numerical values 1, 2, 3, 4 respectively, and the presented 12-item numerical sequence was 121, 342, 314, 324 (Reed & Johnson, 1994). This sequence has unique second-order conditionals (e.g., given 1–2, the next element must be 1), but all first-order conditionals are equally likely. This sequence was repeated eight times in each repeating block. In each random block, all positions were equally likely, with the caveat that no position was repeated twice in a row. This pseudo-randomization assured that on average, there would be no differences between repeating and random blocks in frequency of items or first-order conditionals. Thus, behavioural differences between the blocks could be attributed to participants learning the deterministic second- (or higher-) order structure of the repeating sequence.

Repeating and random blocks were interleaved (Kalra et al., 2019). Participants were first given a random practice block, where the letters corresponding to each key were presented next to each house to facilitate learning of response mappings (Fig. 1). After this training block, participants were given a ten-second break and then the letters were removed and they continued responding. After the training block (1), blocks 2, 4, and 9 were random and the rest (3, 5, 6, 7, 8) were repeating blocks. For a standard measure of SRT learning, we calculated a learning difference score for each participant. The learning score comprised the difference between the mean reaction time (RT) over all valid trials from the final two repeating blocks (7 and 8) and the mean RT over all valid trials from the sequence-flanking random blocks (4 and 9) (see criteria for excluded trials below).

After finishing the last block, participants were queried for explicit knowledge of the sequence. We prompted participants to use the keys to move the star from house to house in the pattern that they had been previously shown. Self-generated sequences were considered evidence of explicit knowledge if they included a correct substring of the standard sequence with a length of 3 or more (e.g., 1–2–1). The length of the longest string of correct consecutive locations was calculated as the explicit learning score for each participant. Only half of the participants in each group were given the explicit knowledge test, due to the concern that the explicit test would bias a subsequent experimental task (not described here).

### Primary SRT Analyses

For exclusionary criteria, we removed trials with errors, delayed RTs (> 1.5 s) and anticipatory RTs (< 100 ms), which we analyzed separately. To control for attention, we excluded participants with fewer remaining trials than the mean across all participants minus two standard deviations. We compared mean RTs and learning scores between groups using an unpaired t-test. A post-hoc power analysis using the R package “pwr” resulted in 90% confidence to detect a difference in learning scores of effect size 0.51 or more.

We then analyzed the errors over the blocks, probing differences using ANOVAs with group as the between-participant factor (i.e. ASD or NT), and block type (i.e., repeating or random) as the within-participant factor. In the random blocks, we analyzed the identity of the errors to look for evidence of sequence-specific errors (Song et al., 2007). If a participant saw ‘1–2–4’ but incorrectly pressed ‘1–2–1’ (i.e., a correct triplet in the standard deterministic sequence), this was considered a sequence-specific error that had a 33% likelihood of happening by chance. We used chi-squared tests to compare the overall group proportions of sequence-specific errors in each block to the hypothesized null proportion of 33%.

### Control SRT Analyses

We conducted analyses to exclude the possibility that third variables were driving group differences in reaction times and errors. We compared the proportion of individuals who had explicit knowledge between the two groups, and used an unpaired t-test to investigate whether there was a mean explicit score difference. In addition, to assure that differences were not driven by IQ or age or sex differences, we used individual matching in the R package ‘MatchIt’ which ensures exact matching for categorical distributions and allows optimization of continuous distributions (Stuart et al., 2011). Lastly, we examined reaction time and error outcomes in the ASD participants with co-occurring ADHD.

## Results

Sixty-six ASD and 71 NT control participants completed the task. Five ASD individuals were excluded for general task inattentiveness (i.e., high error rates), and all analyses were conducted on the remaining 61 ASD and 71 NT participants. Descriptive demographic data are shown in Table 1. Scores on the Autism Quotient were significantly higher in the ASD Group than the NT Group (*t*(130) = 9.48, *p* < 0.001). ﻿There were no significant group differences in age (*t*(130) = 1.70, *p* = 0.09*)* or non-verbal IQ scores (*t*(130) = 1.57, *p* = 0.12) between the groups. In a supplementary analysis, we individually matched the two groups on non-verbal IQ, self-reported sex, and age**.** Results in the individually matched sample were similar (Supplement), and we report results from the full sample here.

### Reaction Times

**Fig. 2:**
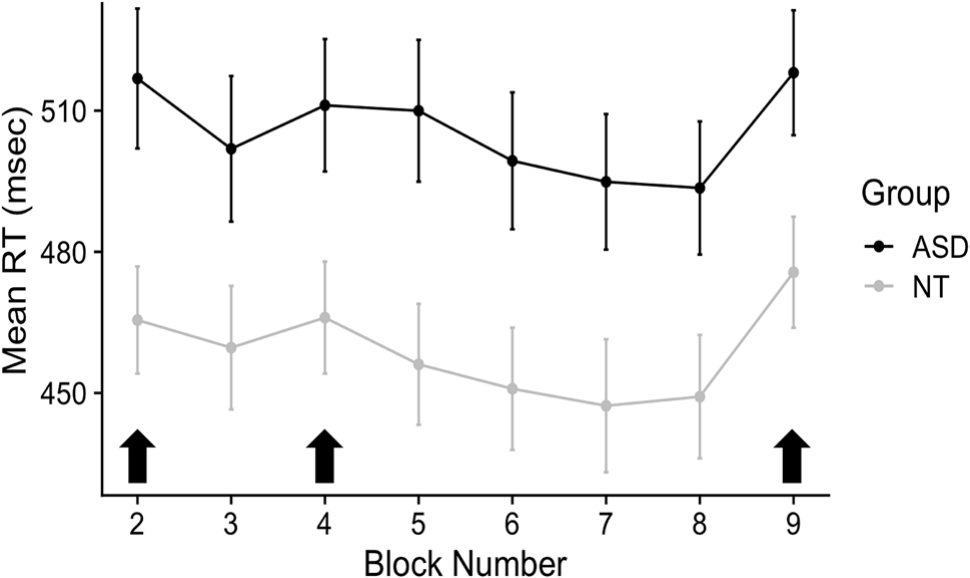
Reaction times in blocks 2–9 in ASD and NT Groups. Mean reaction times (RT) in milliseconds with standard errors are plotted for the two groups. Random blocks (2, 4, 9) are indicated with the black arrows; other blocks (3, 5, 6, 7, 8) are repeating blocks. The NT Group was significantly faster than the ASD Group, but both groups showed evidence of learning with RTs being faster for repeating than random blocks

The ASD Group (M = 510 ms) had significantly slower reaction times (RTs) than the NT Group (M = 460 ms) (*t(*130*)* = 2.89, *p* = 0.004). Both groups showed evidence of sequence-specific learning in RTs, with decreasing RTs across repeating blocks and increasing RTs for random blocks (Fig. 2). There was no significant difference in learning scores between the groups (*t*(123) = 1.11, *p* = 0.13). Because the ASD group was slower overall, we conducted a post-hoc analysis with subgroups of the larger sample by individually matching the two groups on overall mean RTs using ‘MatchIt’, which uses nearest neighbor matching. There was, again, no significant difference between learning scores between the RT-matched NT (N = 48) and ASD (N = 48) groups (*t(*94) = 0.04, *p* = 0.52).

### Errors

**Fig. 3:**
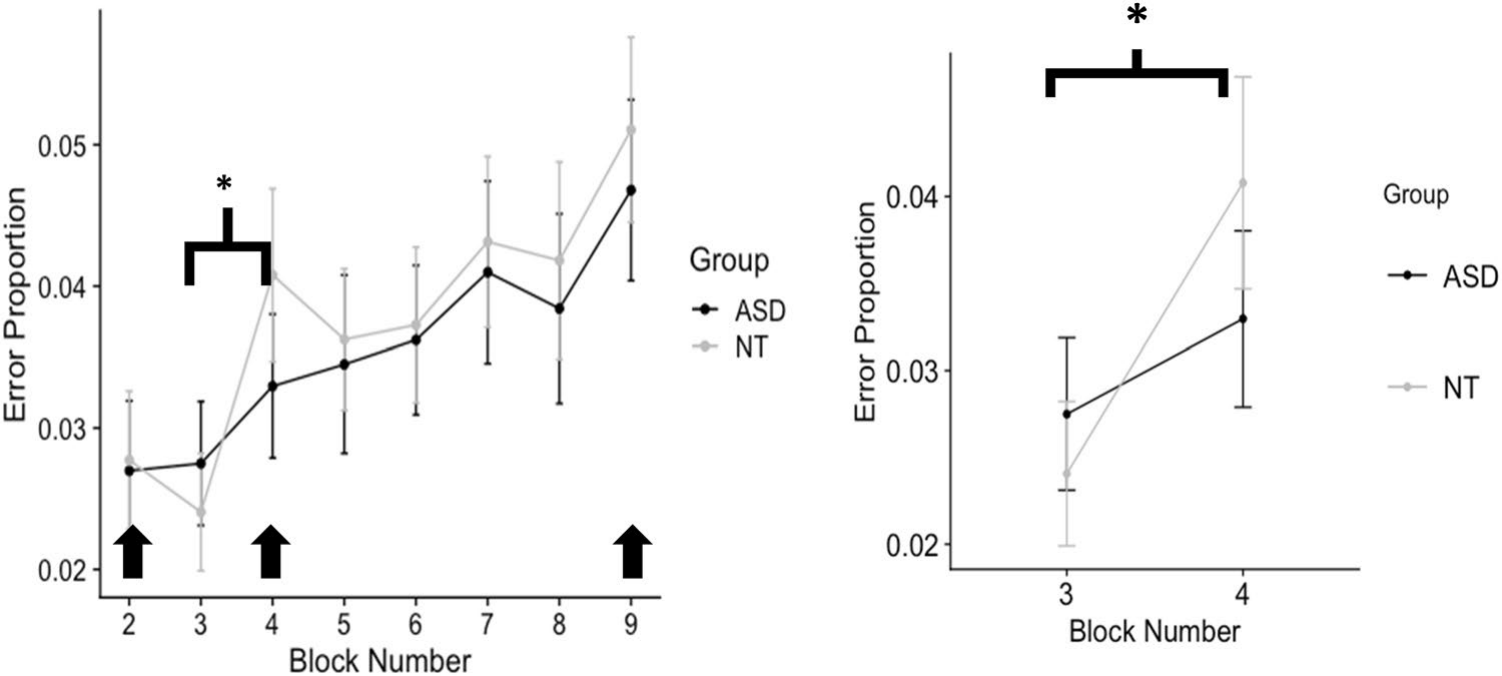
Neurotypical group made more errors in random block 4 after initial presentation of the 12-item repeating sequence in block 3. Mean proportion of errors per group are shown for each block of the experiment; random blocks (2, 4, 9) are indicated with black arrows. In rightmost panel, significant differences in errors within blocks 3, 4 between groups are shown with *p < 0.05

NT (3.8%) and ASD (3.6%) groups had comparable error rates, with no significant difference over all blocks (*t*(130) = 0.43, *p* = 0.67*)*. Both groups showed a significant increase in errors over blocks (F(1,130) = 22.84, *p* < 0.001). There was, however, a significant block (blocks 3, 4, and 5) by group interaction (*F*(2,130) = 4.29, *p* = 0.04). The NT Group made significantly more errors in random block 4 than in block 3 (initial presentation of repeating sequence) (*t*(70) = 5.2, *p* < 0.001), whereas the ASD Group did not make significantly more errors (*t*(61) = 1.6, *p* = 0.06) (Fig. 3).

**Fig. 4:**
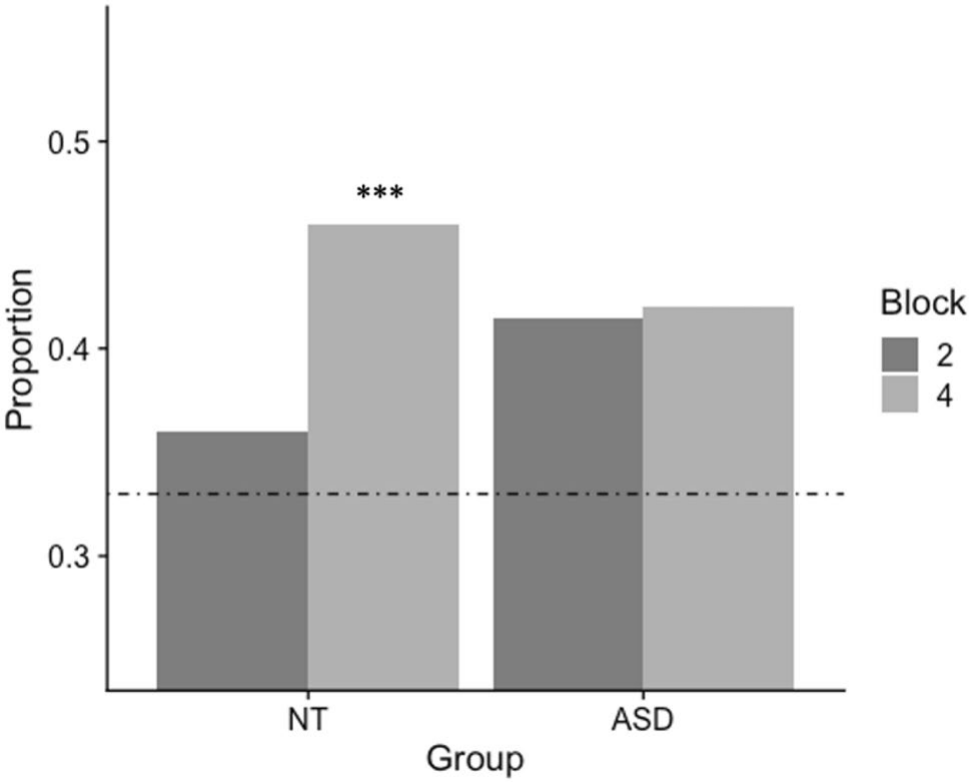
NT individuals show significantly more sequence-specific errors than chance in block 4, whereas ASD individuals do not. The proportions of sequence-specific errors over all errors are shown for each block for each group. Block 2 is before sequence presentation, Block 4 is after the first presentation. Chance levels would predict 33% (1 in 3 chance of picking a second-order transition corresponding to the sequence) shown by broken line. Significance here is shown as chi-square *p* values, where ***p < *0.001*

We tested whether the errors were specifically related to predictions learned during the preceding standard blocks (Fig. 4). In the NT Group, but not ASD Group, the number of sequence-specific errors was significantly higher than chance (33%) in random block 4 (*X*^2^(1,196) = 14.8, *p* < 0.001), although there was not a significant difference between groups (*X*^2^(1,126) = 1.63, *p* = 0.44).

We also tested whether the two groups differed in anticipatory errors, button presses that occurred faster than 100 ms (Fig. S3). The NT Group showed a significantly higher proportion of anticipatory errors in block four than the ASD Group (*t*(130) = 2.04,*p* = 0.02)*.*

**Fig. 5:**
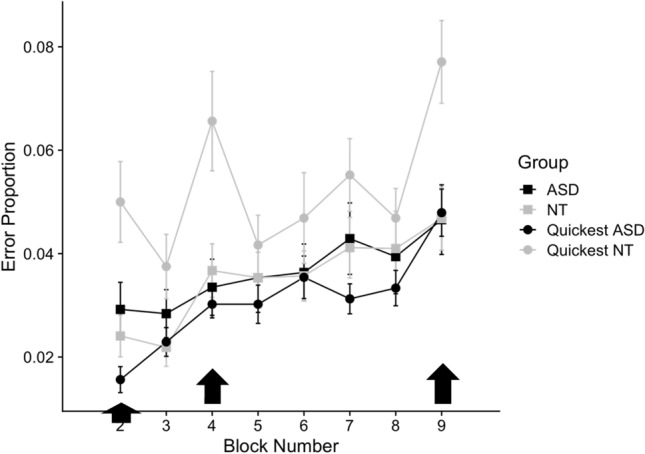
The quickest NT individuals show a pronounced increase in errors after first presentation of the 12-item sequence. The groups shown in this plot are the top ten overall quickest ASD participants (black line with circles), the top ten quickest NT participants (grey line with circles), and the remaining participants in each group (grey and black lines with squares). Trajectories of mean error proportion per group are shown over the blocks of the experiment, random blocks (2, 4, 9) are indicated with the black arrows

We explored whether the error differences between groups might be due to speed-accuracy tradeoffs. When individually matching the groups by RT, the error difference between blocks three and four was still significant (*F*(102,2) = 4.01, *p* = 0.046). We also compared the top ten quickest NT participants (mean RT = 0.36) with the top ten quickest ASD individuals (mean RT = 0.39) (Fig. 5). The quickest NT participants showed a pronounced increase in errors, while the quickest ASD individuals showed little or no increase in errors.

### Explicit Memory for Sequences

We examined whether there were any differences in explicit knowledge of the sequence between the ASD and NT groups (Fig. S4). Note that because the SRT task was counterbalanced with another task (not presented here), only 39 NT and 29 ASD individuals received the explicit knowledge probe. The two groups showed similar proportions of participants who recalled more than three items correctly (NT: 22/39, 56%, ASD: 15/29, 51.7%). Mean explicit scores for ASD (M = 2.48) and NT (M = 2.49) did not differ significantly (*t*(66) = 0.007, *p* = 0.99).

### ADHD Co-occurrence

We also examined whether differences between the ASD and NT groups could have been driven by the 13 participants with ADHD comorbidity in the ASD Group. Participants with ADHD and ASD showed comparable learning scores to the other ASD participants (*t*(57) = 0.6, *p* = 0.55). When removing participants with an ADHD comorbidity, the error difference in blocks three and four between ASD and NT participants remained (*F(*2,117) = 5.9, *p* = 0.016).

## Discussion

In a serial reaction time (SRT) task, the ASD Group had slower overall reaction times, but demonstrated learning scores equivalent to the neurotypical (NT) Group, decreasing their reaction times to the repeated blocks to a similar degree. Additionally, the NT Group made more errors in the first block of random trials following initial exposure to the repeating sequence.

The observation of typical sequence-specific learning in the ASD Group aligns with most previous studies. Multiple studies of SRT have found that learning with repeated exposure to a sequence was comparable between ASD individuals and neurotypical individuals (Barnes et al., 2008; Brown et al., 2010; Izadi-Najafabadi et al., 2015; Nemeth et al., 2010; Rybicki et al., 2021; Travers et al., 2010; Zwart et al., 2017, 2018). Many of these previous studies may have been underpowered to detect a difference (with sample sizes of 15–30); in the present study, there were 61 ASD and 71 NT participants. Further, SRT is one of multiple kinds of procedural memory that have been found to be typical in ASD (Foti et al., 2015).

The finding of slower overall reaction times in ASD is also consistent across SRT studies (Brown et al., 2010; Nemeth et al., 2010; Mostofsky et al., 2000; Travers et al., 2010; Travers et al., 2015). Slowed reaction times in autism have been found across multiple tasks (Morrison et al., 2018). Thus, slower reaction times may reflect broad motor difficulties in autism (Gowen & Hamilton, 2013).

When examining error outcomes, the NT and ASD groups diverged for the first block of random trials after initial exposure to the sequenced trials. A significant proportion of those errors in the NT Group were sequence-specific, e.g., a participant saw ‘1–2–4’ but incorrectly pressed ‘1–2–1’ (a correct triplet in the repeated deterministic sequence). In addition, the proportion of anticipatory (< 100 ms) errors was higher in the NT Group than the ASD Group. This difference was not due to differing speed-accuracy tradeoffs between the groups. The error difference remained after individually matching participants across NT and ASD groups by overall RT. This difference, however, did not persist after the initial transition from random to sequenced trials. After many contiguous sequenced trials (blocks 5–8), the transition to the final random block was similar across the two groups. Also notable in both groups was an overall increase in errors over blocks, which taken in tandem with decreases in reaction time, could suggest fatigue or overconfidence. Speculatively, this could relate to the self-administration of the online experiment in which no experimenter was present to encourage focus.

Why was this error difference between groups specifically present in the early phase of learning? Early in the experiment, participants are presented with a random block, a sequenced block, and then another random block. This on/off pattern may have made ASD individuals less confident in what they learned, as the environment was more ‘volatile’. This would be in keeping with theories of environmental volatility in autism (Lawson et al., 2017), as well as one previous SRT study which used alternating random and sequenced blocks and found decreased learning in autism (Travers et al., 2015).

The early differences in errors could be interpreted in two different ways, as either an example of enhanced rationality in ASD or diminished utilization of predictions in ASD. In the domain of judgment and decision making, ASD individuals often out-perform their neurotypical counterparts by avoiding the use of misleading biases and heuristics that are common in neurotypical individuals (Rosenkrantz et al., 2021). By this view, the NT Group misleadingly employed explicit memory to anticipate the next random trial location, which increased both anticipatory errors and response errors. The ASD Group, on the other hand, exhibited enhanced rationality (i.e., more accurate performance) by not employing explicit memory of the sequence. It is possible that the early difference in errors was due to the way explicit memory was employed by the NT Group, even though explicit memory for the sequence did not differ significantly between the groups. This may represent a strength for autistic individuals; during the early phase of learning, ASD individuals present with more veridical responses and a reduced employment of prior knowledge, resulting in fewer errors.

Alternatively, the error difference may have reflected differences in predictive processes. A systematic review (Cannon et al., 2021) found evidence that ASD is associated with reduced spontaneous engagement of predictive motor processes, including gaze (Barzy et al., 2019; Greene et al., 2019; Schuwerk et al., 2016) and object interception (Ament et al., 2015; Landa et al., 2016). In a reaction time task with partial predictability of targets, ASD participants showed reduced modulation of reaction times by predictability, while EEG recordings of ASD participants showed reduced predictive mu rhythm desynchronization and reduced predictive ramping potentials (Thillay et al., 2016). In Thillay et al., all participants were explicitly instructed of the rules by which some targets could be predicted; therefore, these results do not support a deficit in learning predictive patterns, but instead suggest a lack of engagement of preparatory motor processes by predictability. These preparatory processes may be responsible for the sequence-specific errors in the NT population in the current work, and reduced engagement of these processes in ASD might explain the relative lack of such errors. It would be interesting to more fully characterize the preparatory processes that are under-utilized in ASD.

If people with ASD are indeed less predisposed to utilize predictive cues in motor behavior, this suggests specific pathways by which certain social symptoms of ASD may emerge, along the lines discussed in Northrup (2017). For example, social cues may evoke less engagement of the gaze in infants predisposed to ASD, leading to a cycle of reduced social engagement and reduced social learning throughout childhood and into adulthood. This perspective could potentially inform early interventions to support social learning in infants showing early evidence of ASD.

A difference in prediction could have consequences not just in social behavior but in daily life. Studies have found that autistic individuals have an attenuated ability to predict actions and object locations (Ganglmayer et al., 2020; Greene et al., 2019). Challenges with action and location prediction could have negative consequences across many tasks of daily living, such as crossing the street, driving a car, or riding a bike, as these skills require frequent predictions (e.g., related to the actions and locations of pedestrians, drivers, and roadside hazards).

Two limitations of the present study may be noted. First, only half of the participants received the test of explicit memory for the sequence, but the typical learning was seen in the full sample of participants. Second, the present results were obtained from ASD individuals with normal-to-high nonverbal IQ, and may therefore not be reflective of the entire autism spectrum.

## Conclusion

In this preregistered, large-sample online study of SRT, we found no difference in learning between autistic and neurotypical adults. These results challenge the notion that there is broadly atypical predictive learning in autism. However, there was a small, temporally limited decrease of sequence-specific errors in the ASD adults that may be interpreted as enhanced rationality or differences in predictive motor preparation.

### Supplementary Information

Below is the link to the electronic supplementary material.

Supplementary file1 (DOCX 166 kb)

## Data Availability

Data and code will be made publicly available on publication at OSF. https://osf.io/4g37d/?view_only=b13b9875d0174577af3d3b74ee059234
