# Supplementary material for: Autistic Adults Show Intact Learning on a Visuospatial Serial Reaction Time Task
Source: J Autism Dev Disord. 2023 Jan 14;54(4):1549–57. doi: 10.1007/s10803-023-05894-y (PMC10981634; doi:10.1007/s10803-023-05894-y)
Supplement: Supplementary file 1 — Supplementary file1 (DOCX 166 kb) [file 10803_2023_5894_MOESM1_ESM.docx]

**Autistic adults show intact learning on a visuospatial serial reaction time task**

**Supplemental Table 1**

| **Study** | **Task** | **Sample sizes** | **Group difference** | **Effect Size****, if available** |
| --- | --- | --- | --- | --- |
| Mostofsky 2000 | SRT | 17 NT/ 11 ASD | Yes | -1.82 |
| Smith 2003* | SRT | 23 NT/ 17 ASD | No |  |
| Muller 2004 | SRT | 8 NT/ 8 ASD | No | 0.04 |
| Gordon Stark 2007a | SRT | 9 NT/ 7 ASD | Yes |  |
| Gordon Stark 2007b | SRT | 5 NT / 5 ASD | No | 0.12 |
| Barnes 2008 | ASRT** | 14 NT/ 14 ASD | No | 0.14 |
| Brown 2010 | ASRT** | 31 NT / 31 ASD | No | 0.27 |
| Nemeth 2010 | ASRT** | 13 NT/ 13 ASD | No | -0.61 |
| Travers 2010 | SRT | 18 NT/ 15 ASD | No | 0.40 |
| Travers 2015 | SRT | 15 NT/15 ASD | Yes |  |
| Izadi-Najafabadi 2015 | SRT | 32 NT/ 30 ASD | No |  |
| Sharer 2015 | SRT | 36 NT/ 17 ASD | No |  |
| Zwart 2017 | SRT | 20 NT/ 20 ASD | No |  |
| Zwart 2018a | SRT | 17 NT/ 16 ASD | No |  |
| Zwart 2018b | SRT | 37 NT/ 35 ASD | No |  |
| Rybicki 2021 | SRT | 35 NT/ 28 ASD | No |  |
| Ward 2021 | SRT | 25 LL ASD/ 28 HL ASD*** | No |  |

*Unpublished thesis.

**The alternating serial reaction time task, a version of the SRT where probabilistic deviants are interspersed in the sequence. ﻿(Howard & Howard, 1997).

*** Ward et al 2021 conducted a study with two groups of three-year-olds. Those with siblings with autism were considered high-likelihood (HL) of an autism diagnosis, and those without siblings with autism were considered low-likelihood (LL).

**** Effect sizes are reported here as standardized mean differences, where more negative values favor the control group.

**Supplement: Results**

**Individual matching:** After individually matching on non-verbal IQ , self-reported sex, and age, 43 participants were left in each group. The adjusted quartile distributions are shown below, along with the mean reaction times per block. Mean learning scores were not significantly different between groups (*t(*84) = -0.357, *p =* 0.72).


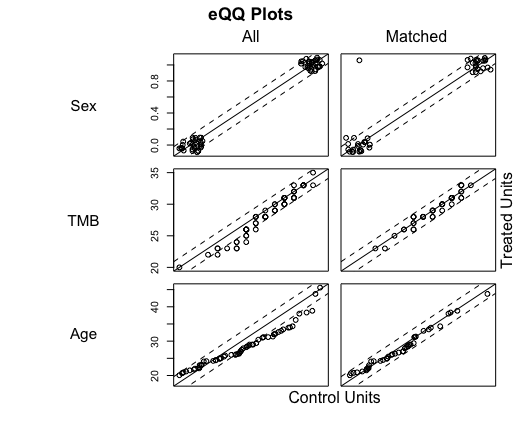


**Fig S1** Quartile distributions. Matched sample, on right, shows more balance.


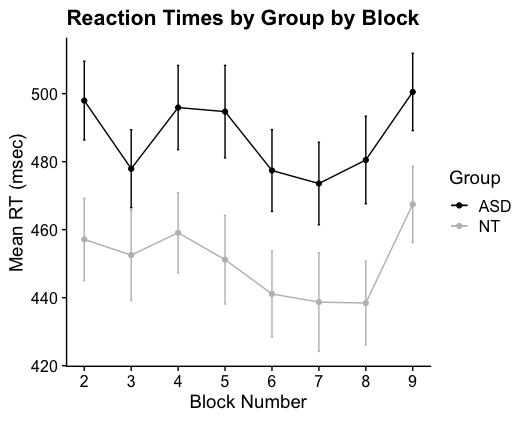


**Fig S2** Reaction times in blocks 2-9 in matched ASD and NT Groups. Mean reaction times (RT) in milliseconds with standard errors are plotted for the two groups. Random blocks (2,4,9) are indicated with the black arrows; other blocks (3,5,6,7,8) are repeating blocks. The NT Group was significantly faster than the ASD Group, but both groups showed evidence of learning with RTs being faster for repeating than random blocks.


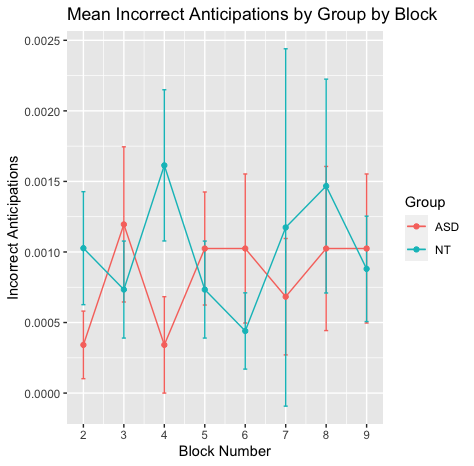


*****

**Fig S3** Incorrect anticipations (IA) are reactions to the wrong item that are faster than 100 ms. Trajectories of mean IA proportion per each group are shown over the blocks of the experiment, random blocks (2,4,9) are indicated with the black arrows. Neurotypicals show significantly more incorrect anticipations in block 4, the first rebound block.


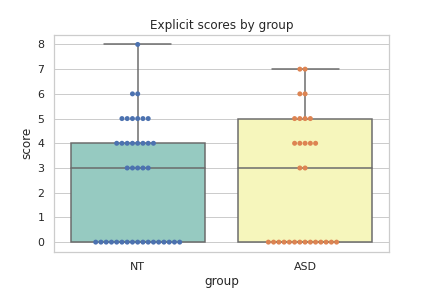


**Fig S4** Plotted are boxplots of the explicit scores for those individuals given the explicit test after the task. A score of 3 represents successful recall of one triplet of locations from the sequence.
